# The odor of Osmanthus fragrans attenuates food intake

**DOI:** 10.1038/srep01518

**Published:** 2013-03-22

**Authors:** Takashi Yamamoto, Tadashi Inui, Tadataka Tsuji

**Affiliations:** 1 Department of Health and Nutrition, Faculty of Health Science, Kio University, 4-2-4 Umami-naka, Koryo, Kitakatsuragi, Nara 635-0832, Japan; 2 Division of Behavioral Physiology, Department of Behavioral Sciences, Graduate School of Human Sciences, Osaka University, 1-2 Yamadaoka, Suita, Osaka 565-0871, Japan; 3 1^st^ Department of Oral and Maxillofacial Surgery, Graduate School of Dentistry, Osaka University, 1-8 Yamadaoka, Suita, Osaka 565-0871, Japan

## Abstract

Odors have been shown to exert an influence on various physiological and behavioral activities. However, little is known whether or not odor stimulation directly affects the levels of feeding-related neuropeptides. Here we show that the neural transmission by Osmanthus fragrans (OSM) decreased the mRNA expression of orexigenic neuropeptides, such as agouti-related protein, neuropeptide Y, melanin-concentrating hormone and prepro-orexin, while increased anorexigenic neuropeptides, such as cocaine- and amphetamine-regulated transcript and proopiomelanocortin in rats. The decreased number of orexin-immunoreactive neurons in the hypothalamus coincided well with the OSM-induced decreases in the expression of prepro-orexin mRNA. This study demonstrates that the OSM odor, which is known to have a mild sedative effect, decreases the motivation to eat, food intake and body weight, accompanied by sluggish masticatory movements. The data suggest that these effects are due to suppression of orexigenic neuropeptides and activation of anorexigenic neuropeptides in the hypothalamus.

Odors affect and/or induce various physiological and mental activities depending on their qualities and hedonic tones^1^,^2^,^3^,^4^,^5^. As far as food intake is concerned, olfaction plays the dominant role in sensing the flavors of the foods^6^. Feeding behavior is necessary to maintain energy homeostasis through a series of processes such as the pre-ingestive appetite, chewing/drinking and intake, with the experience derived from the foods exerting an impact on both post-ingestive digestion and absorption^7^. Although it is reported that fasting increases and satiation decreases olfactory detection in rats^8^,^9^, essentially no systematic investigation has been performed to elucidate how olfactory stimuli affect each aspect of feeding behavior. It is reported that olfactory stimulation with the scent of grapefruit or lavender oil exerts effects on the sympathetic and parasympathetic nervous system, enhancing and suppressing lipolysis through a histaminergic response, resulting in a reduction and enhancement of appetite and body weight, respectively^10^,^11^.

Our research group has been studying the functions of orexigenic neuropeptides for some time, and reported that the mRNAs for the orexins and neuropeptide Y (NPY) are inducibly expressed by palatable taste stimuli^12^, and also that intracerebroventricular administrations of these neuropeptides increased saccharin intake^13^, gastric motility^14^ and ingestive behavior, along with enhanced masticatory muscle activity^15^. Although a recent study^16^ has reported that a pleasant odor stimulation with linalool which is identified in numerous foods and flowers regulates the gene expression related to the functions of neuronal developments in the hypothalamus of rats under a restraint-stressed condition, little is known whether or not odor stimulation directly affects the levels of orexigenic and anorexigenic neuropeptides so as to control food intake. This topic is largely unexplored and thus an important research target, in spite of the fact that the central olfactory system itself including sensory cells in the olfactory epithelium and neurons in the olfactory bulb have been intensely investigated^17^,^18^ since Buck and Axel^19^ first reported a diverse family of odorant receptors in 1991.

The beverages taken before, during and/or after meals have certain characteristic odors as well as tastes. To explore the effects of such odors on the hypothalamic feeding center, we selected the odors of milk, coffee, vanilla, cocoa, hops (beer) and Osmanthus fragrans (OSM, fragrant tea olive) as representative drinks and/or desserts across the world. OSM is an evergreen shrub which is grown in Eastern Asia and its flowers are used in tea and wine, which are consumed on a daily basis in Taiwan and China^20^. In our preliminary study, which was previously reported as a brief abstract^21^, we screened the above 6 odors on the basis of the level of prepro-orexin mRNA after odor stimulation, since we previously had reported that orexins play an important role in taste-induced over-consumption^12^. The result indicated that the odor of milk tended to increase the prepro-orexin mRNA, but that of OSM decreased it, while the other odors tested exerted no particular effects.

The present study, therefore, sought to confirm these previous findings and to investigate the mechanism of the effects of OSM odor and milk odor, as possible counterparts, on feeding behavior based on the results from molecular biological, immunohistochemical, electrophysiological and behavioral investigation.

## Results

### Prepro-orexin mRNA expression

**Figure 1:**
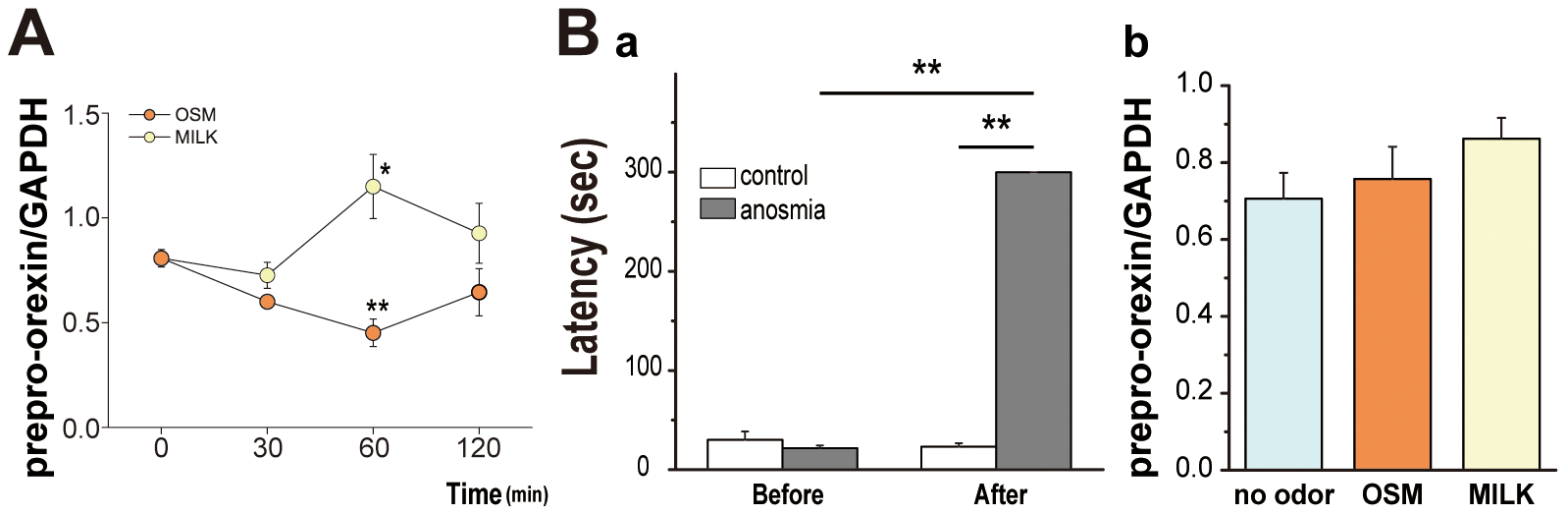
Effects of odors on the expression of prepro-orexin mRNA in normal and anosmic rats. A, The time course of the expression of prepro-orexin mRNA after the onset of odor stimulation. The expression was increased and decreased by the odors of milk and OSM, respectively. Data are the means ± SE (n = 5 or 6 per each group). B, The effects of odors on the expression of prepro-orexin mRNA after anosmic treatment: a, comparison of the latency to the finding of the scented mash before and after the anosmic treatment in the control and anosmic groups. Data are the means ± SE (n = 12 per each group); b, the expression of prepro-orexin mRNA after odor stimulation in anosmic rats. The odor of OSM or milk did not exert any effect in the anosmic rats on the level of prepro-orexin mRNA. Data are the means ± SE (n = 4 per each group). *P < 0.05; **P < 0.01.

As a first step to investigate our previous findings, we examined the effects of the odors of OSM and milk on the prepro-orexin mRNA level. Fig. 1A shows the time course of the expression of prepro-orexin mRNA in hypothalamic specimens taken at different times after the onset of odor stimulation. The expression was gradually decreased by the OSM odor and increased by the milk odor such that a statistically significant difference was evident 60 min after the onset of stimulation in comparison with the pre-stimulus level. A one-way ANOVA showed the main effects over time with OSM [F(3,22) = 4.57, *p* < 0.05] and milk [F(3,20) = 3.21, *p* < 0.05]. Post hoc analysis revealed that significant differences were obtained between the pre-stimulus level and the level after 60 min for both OSM (*p* < 0.01) and milk (*p* < 0.05).

### Effects of anosmia

To examine whether the altered mRNA levels were induced by olfactory neural information transmitted from olfactory receptors to the brain, we compared the mRNA expression level between normal and anosmic rats. Fig. 1Ba shows that the anosmic treatment carried out by damaging olfactory cells with zinc sulfate solution applied into the nasal cavity was effective, since those rats exhibited a significantly longer latency to finding the scented mash hidden in the sawdust than the control rats. The Wilcoxon signed-ranks test showed a significant difference between the anosmic and control groups after surgery (i.e. the anosmic procedure) (Z = 2.52, *p* < 0.05), and before and after surgery in the anosmic group (Z = 3.29, *p* < 0.01). After anosmia was performed, no changes occurred in the prepro-orexin mRNA level examined 60 min after the odor stimulation (Fig. 1Bb) [F(2,17) = 1.19, *p* = 0.33], indicating that the odor effects were induced by olfactory neural messages.

### mRNA expression of feeding-related neuropeptides

**Figure 2:**
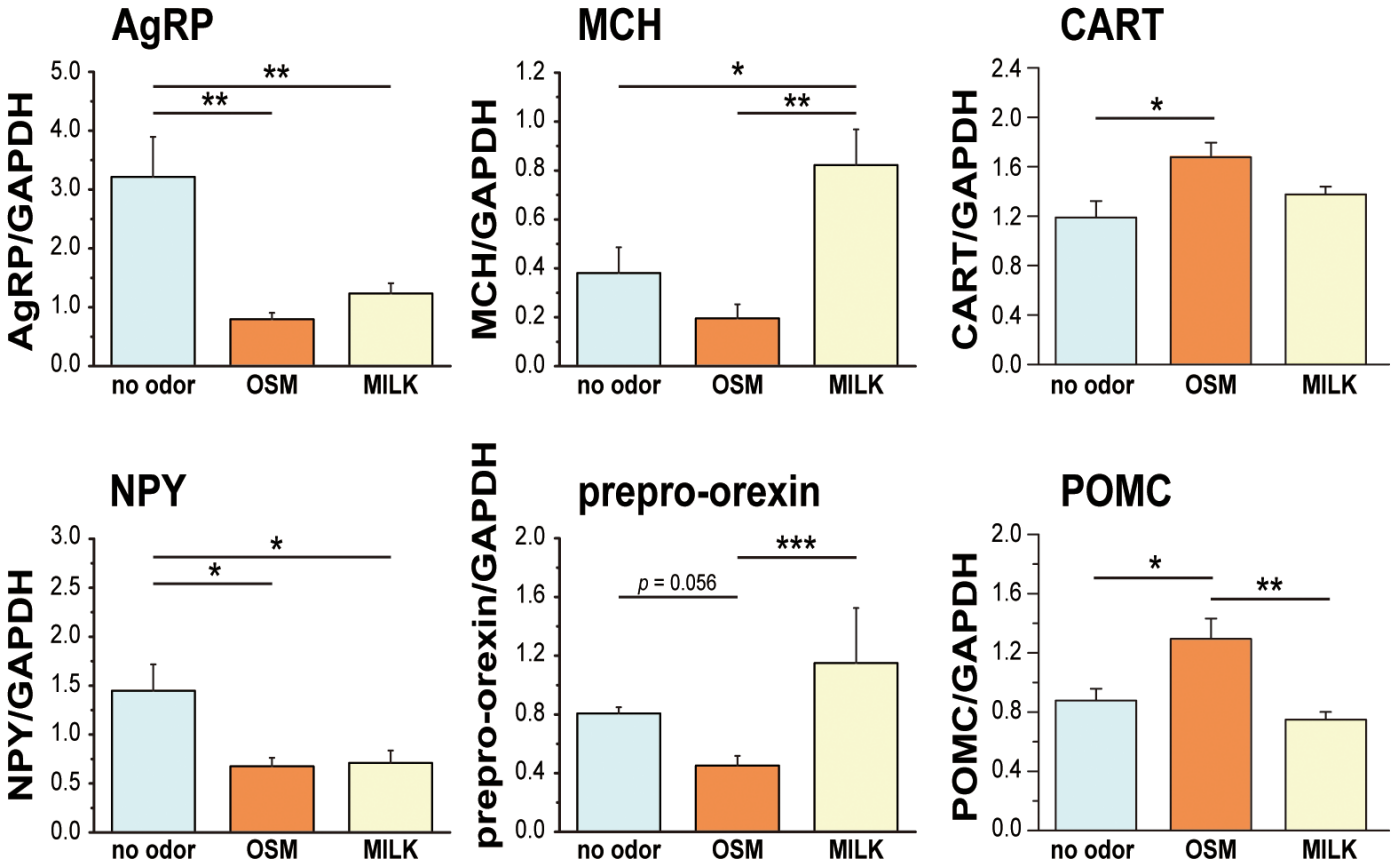
Effects of odors on the expression of mRNAs for feeding-related neuropeptides in the hypothalamus. Comparison of the effects of odor stimulation on the mRNA expression of orexigenic neuropeptides, AgRP, NPY, MCH and prepro-orexin, and anorexigenic neuropeptides, CART and POMC. Data are the means ± SE (n = 6 per each odor condition). *P < 0.05; **P < 0.01, ***P < 0.001.

To examine the effects of these odors on the expression of hypothalamic orexigenic and anorexigenic neuropeptides, the mRNAs for agouti-related protein (AgRP), melanin-concentrating hormone (MCH), NPY, prepro-orexin, cocaine and amphetamine regulated transcript (CART) and proopiomelanocortin (POMC) were measured using the RT-PCR method for the hypothalamic specimens taken 60 min after the onset of stimulation. The OSM odor decreased the expression of the mRNA level for all four of the orexigenic neuropeptides, AgRP, MCH, NPY and prepro-orexin (Fig. 2). The effects of the milk odor differed depending on the neuropeptides, i.e. it increased the mRNA for MCH and prepro-orexin in the lateral hypothalamus, while it decreased the mRNA for AgRP and NPY expressed in the hypothalamic arcuate nucleus. On the other hand, the OSM odor increased the expression of the mRNA level for the anorexigenic neuropeptides, CART and POMC, while the milk odor induced little effects (Fig. 2). A one-way ANOVA showed the main effect of OSM to be on AgRP [F(2,14) = 11.75, *p* < 0.01], MCH [F(2,13) = 8.17, *p* < 0.01], NPY [F(2,13) = 6.09, *p* < 0.01] and prepro-orexin [F(2,14) = 13.35, *p* < 0.01], CART [F(2,14) = 4.85, *p* < 0.05] and POMC [F(2,14) = 8.62, *p* < 0.01]. Post hoc analysis revealed significant differences between the control and OSM for AgRP (*p* < 0.01), NPY (*p* < 0.05), CART (*p* < 0.05) and POMC (*p* < 0.05), and between control and milk for AgRP (*p* < 0.01), MCH (*p* < 0.05) and NPY (*p* < 0.05).

### Expression of orexin in the neurons of the hypothalamus

**Figure 3:**
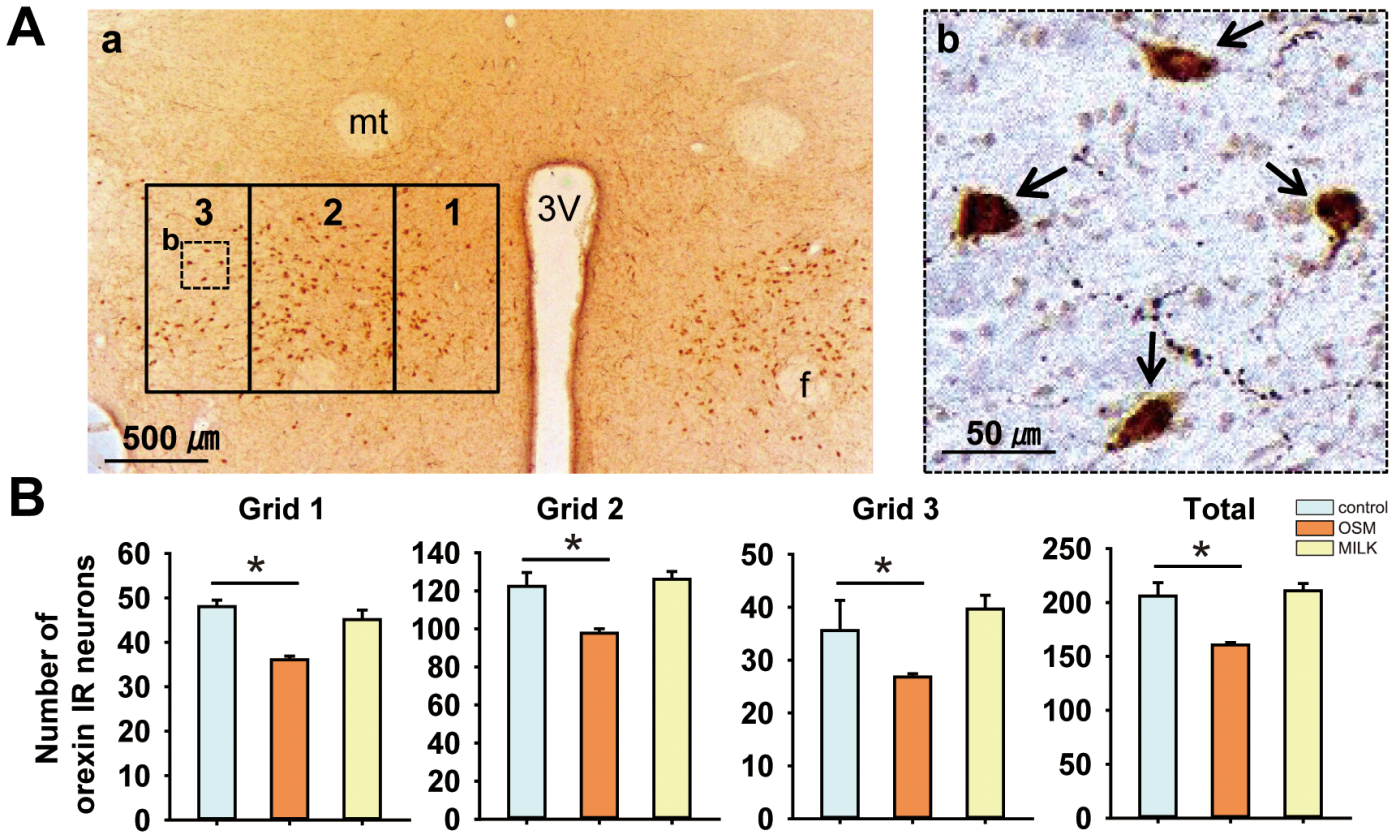
Effects of odors on the number of orexin neurons in the hypothalamus. A, Localization of the orexin-immunoreactive (orexin-IR) neurons in the rat hypothalamus after OSM odor stimulation: a, placement of the medial (grid 1), central (grid 2) and lateral (grid 3) counting boxes in the hypothalamus; b, an enlargement of the boxed area in A with arrows indicating orexin-IR neurons; *3 V*, third ventricle; *f*, fornix; *mt*, mammillothalamic tract. B, The mean (± SE) total number of orexin-IR neurons in each of the three grids in the lateral hypothalamic area for the control, milk and OSM rats. Data are the means ± SE (n = 4 per each odor condition). *P < 0.05 (Mann-Whitney U test).

It is important to determine whether the changes in the mRNAs for the orexigenic neuropeptides are reflected in the production of the relevant neuropeptides. We therefore measured the number of orexin-neurons in the hypothalamus with an immunohistochemical method for the brain taken 60 min after the onset of stimulation. The number of orexin-immunoreactive neurons was counted in each of the medial, central and lateral areas in the lateral hypothalamus (Fig. 3Aa). The number of orexin-immunoreactive neurons in each area was significantly decreased by the OSM odor in comparison with the non-odor controls (Fig. 3B). The milk tended to increase the number of neurons in the central and lateral areas, but the difference was not significant (Fig. 3B).

### Ingestive behavior and body weight with chronic exposure

**Figure 4:**
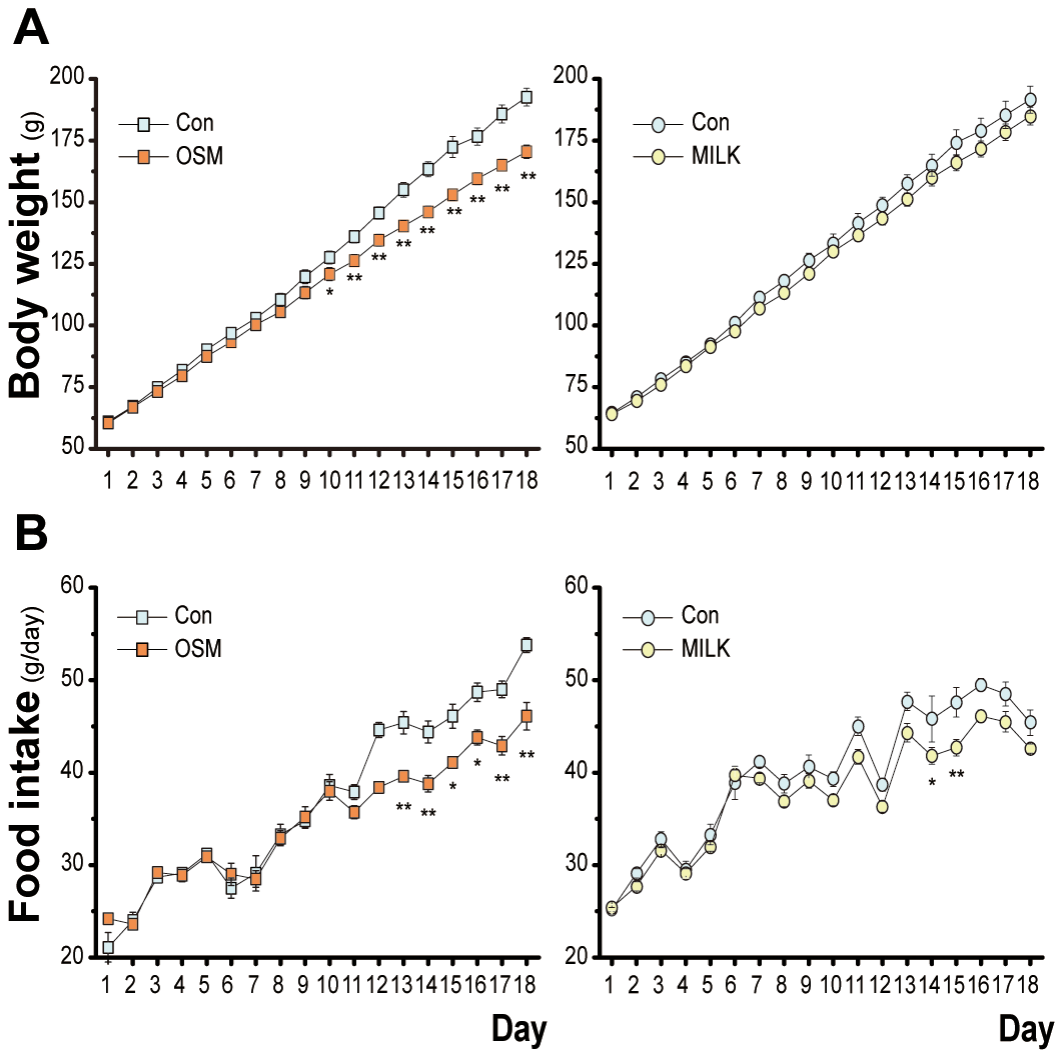
Effects of the odors on body weight and food intake. A, Effects of chronic exposure to the odors on body weight. B, Effects of chronic exposure to the odors on food intake. Data are the means ± SE (n = 6 per each group).

To examine the effects of chronic exposure to these odors on body weight, food intake and water intake, rats were given flavored mashed food, i.e. a mixture of powdered chow with flavored water. We examined the effects of the two odors on food intake and body weight in a long-term exposure experiment in which food with the OSM odor, or the milk odor or without these odors was presented to each of three groups of rats. When the experiment started in 4-week old rats having a body weight of approximately 60 g, the body weight increased without any obvious difference between the OSM rats and control rats and a significantly lower body weight was observed after the 10^th^ day in the OSM group, whereas no significant difference was observed in the milk group (Fig. 4A). With the OSM odor, a two-way (group × day) ANOVA revealed the significant main effects by the group [F(1,11) = 7.72, *p* < 0.05] and day [F(17,187) = 2294.94, *p* < 0.01]. The group × day interaction was also significant [F(17, 187) = 21.86, *p* < 0.01]. Post hoc analysis revealed a significant difference between the OSM and control groups (Day 10, *p* < 0.05; Day 11–18, *p* < 0.01). With the milk odor, a two-way ANOVA revealed the main effect only of the day [F(17, 187) = 1774.48, *p* < 0.01].

The food intake was reduced in the OSM group after the 13^th^ day, but in the milk group the food intake was essentially the same as that in the control group except on the 14^th^ and 15^th^ days, when the milk rats ate less than the control rats (Fig. 4B). With the OSM odor, a two-way (group × day) ANOVA showed significant main effects of both the group [F(1,11) = 12.72, *p* < 0.01] and day [F(17,187) = 177.93, *p* < 0.01]. The group × day interaction was also significant [F(17, 187) = 6.79, *p* < 0.01]. Post hoc analysis revealed a significant difference between the OSM and control groups (Day 15–16, *p* < 0.05; Day 13–14 and 17–18, *p* < 0.01). In addition, with the milk odor, a two-way ANOVA also showed there to be significant main effects of groups [F(1, 11) = 7.35, p < 0.05] and day [F(17, 187) = 176.03, *p* < 0.01]. The group × day interaction was also significant [F(17, 187) = 2.11, *p* < 0.01]. Post hoc analysis revealed a significant difference between the OSM and control group (Day 14, *p* < 0.05; Day 15, *p* < 0.01).

### Chronic exposure to the OSM odor in *ob/ob* mice

**Figure 5:**
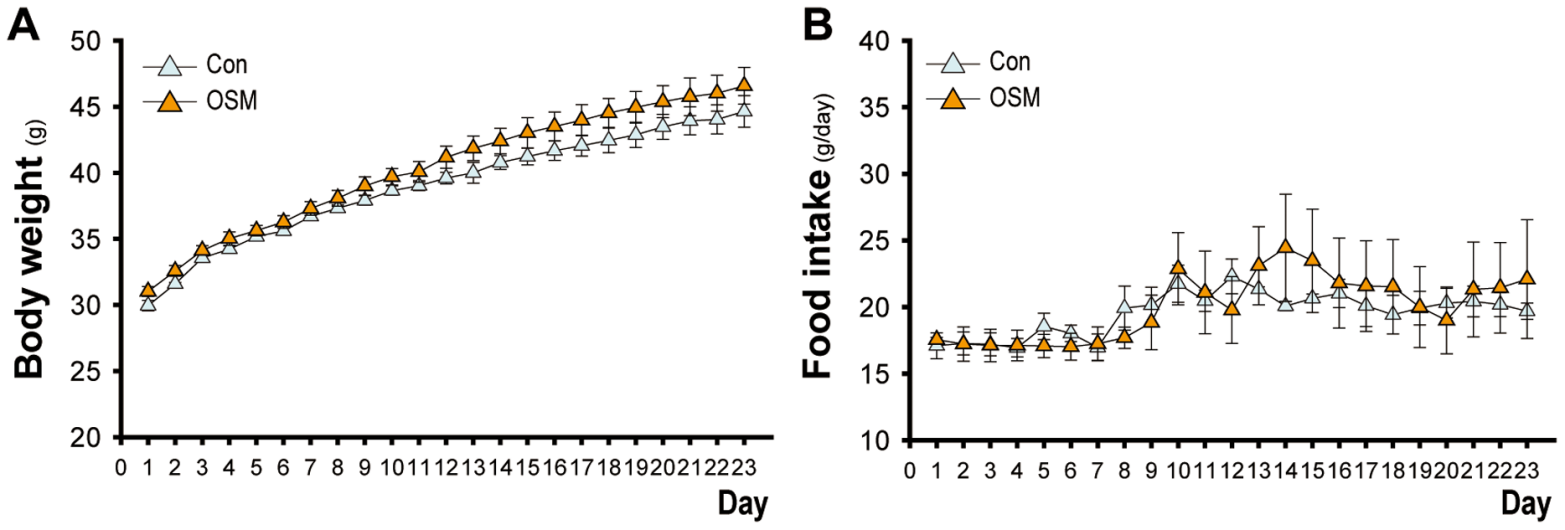
Effects of the OSM odor on body weight and food intake in *ob/ob* mice. A, Effects of chronic exposure to the odor on body weight. B, Effects of chronic exposure to the odors on food intake. Data are the means ± SE (n = 4 per each group).

Essentially the same behavioral experiment was performed with the odor of OSM in *ob/ob* (leptin-deficient) mice where the high level of AgRP, NPY, MCH and orexin and the low level of CART and POMC area known. As shown in Fig. 5A, when the experiment started in 5-week old mice having a body weight of approximately 30 g, the body weight increased with a tendency of gaining more weight in the *ob/ob* mice exposed to the OSM odor than the *ob/ob* control mice. This difference, however, was not statistically significant. A two-way (group × day) ANOVA revealed main effect only of the day [F(22, 126) = 212.70, *p* < 0.001]. The group × day interaction was not significant. The food intake in the OSM group was essentially the same as that in the control group although the increasing pattern was not monotonic (Fig. 5B). A two-way (group × day) ANOVA revealed main effect only of the day [F(22, 132) = 3.81, *p* < 0.001]. The group × day interaction was not significant.

### Ingestive behavior with acute exposure

**Figure 6:**
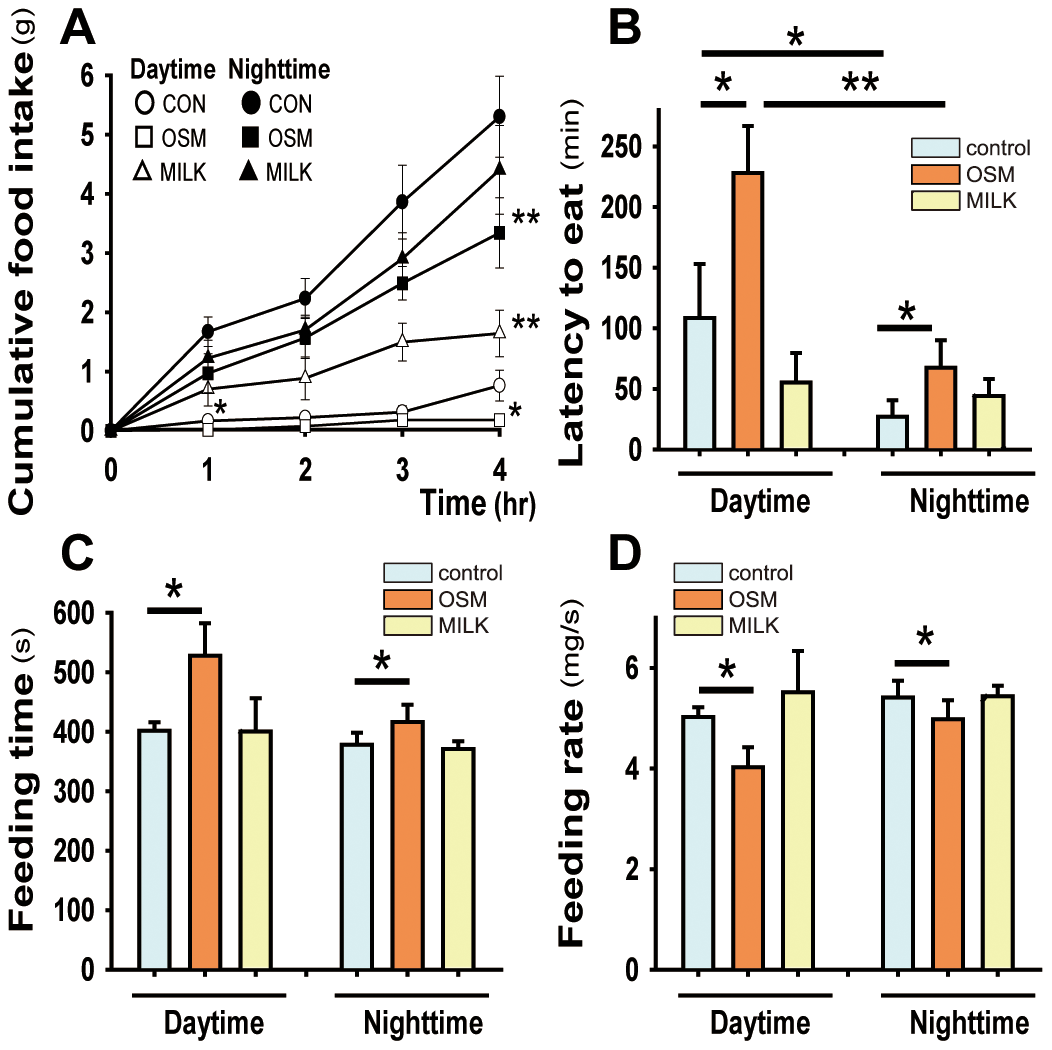
Effects of acute exposure to the odors on feeding behavior during the daytime and nighttime. A, Cumulative food intake during a 4-h long period of free access to pellets; B, Latency to eat; C, Feeding time for the consumption of 2 g of pellets; D, the feeding rate calculated by dividing 2 g of pellets by the feeding time. Data are the means ± SE (n = 8 per each group). *P < 0.05; **P < 0.01 (comparison of the odor conditions in the daytime and nighttime; Wilcoxon signed-ranks test, comparison between the daytime group and nighttime group; Mann-Whitney U test.)

To evaluate the manner in which the rats exposed to the odors ate food, we analyzed the feeding pattern under acute exposure to the OSM or milk odors in both the daytime and nighttime periods using a video camera. The cumulative food intake over 4 hrs in both the daytime and nighttime periods was significantly decreased in the rats exposed to the OSM odor than the milk odor or no odor exposure, and the reverse was true for the milk rats, although a significant difference was detected only in daytime (Fig. 6A). It is noted that the amount of intake was obviously larger in the nighttime than daytime. The latency to the start of eating was significantly prolonged in the OSM rats compared with the control rats (Fig. 6B). The latency was generally longer in the daytime than nighttime. The feeding time for 2 g food was longer in the OSM than control rats (Fig. 6C). Accordingly, the calculated feeding rate (the weight of the pellets/the feeding time) was significantly lower in the OSM rats (Fig. 6D). It is interesting that there was essentially no difference in the feeding time or feeding rate between the daytime and nighttime.

### Masticatory muscle activity

**Figure 7:**
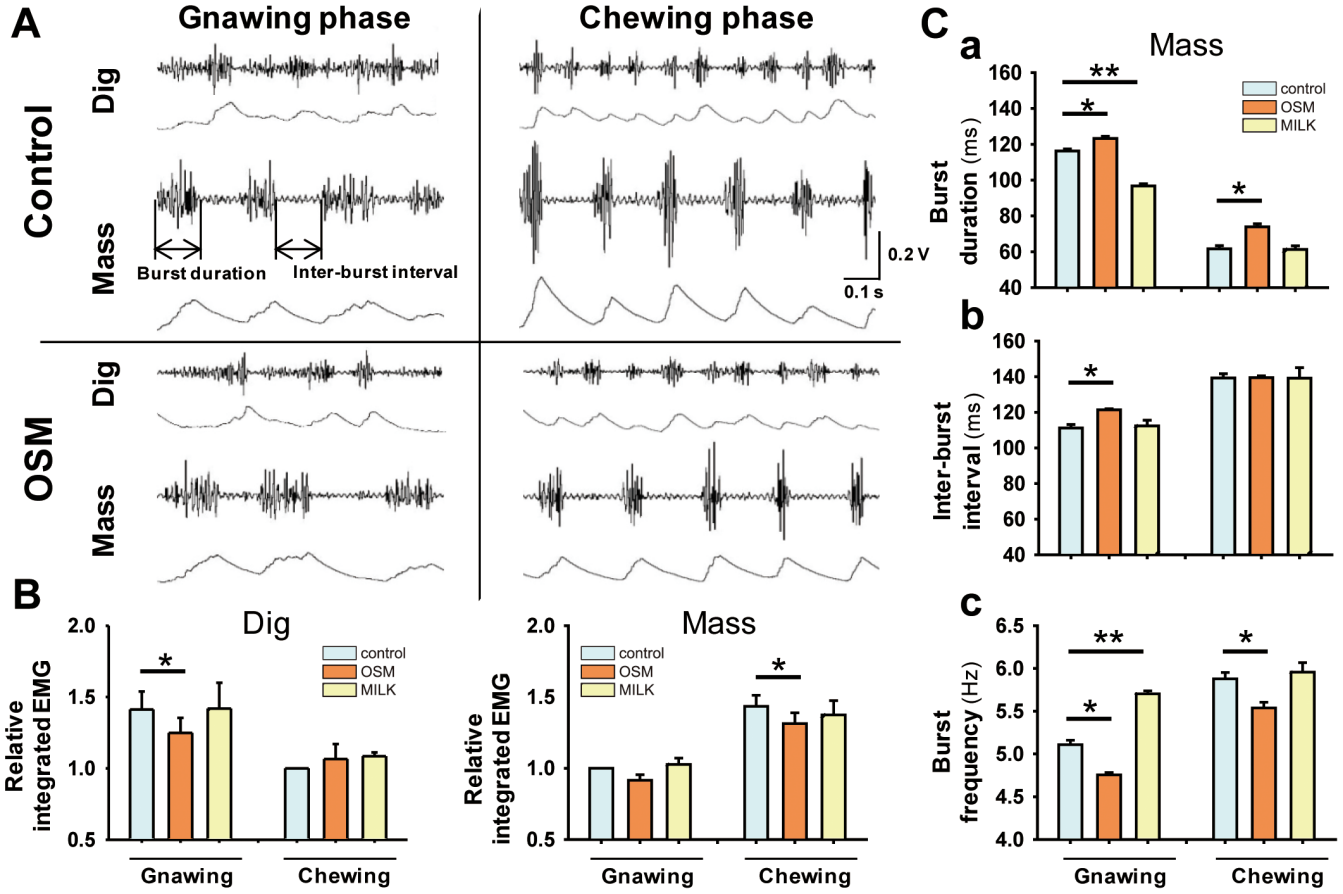
Analyses of masticatory muscle activity under the odor conditions. A, Representative actual electromyography (EMG) recordings and integrated EMG of the digastric and masseter bursts during the eating of the pellets under the no odor (control) and OSM odor conditions. B, Comparison of the relative integrated EMG of the digastric and masseter muscles in the gnawing and chewing phases among the control, milk and OSM odor conditions when the integrated EMG in the control chewing phases was taken as 1.0. Data are the means ± SE (n = 6 per each group). *P < 0.05 (Wilcoxon signed rank test). C, Comparison of the parameters of the bursts of the masseter muscle in the gnawing and chewing phases among the control, milk and OSM odor conditions: a, burst duration; b, inter-burst interval; c, burst frequency. Data are the means ± SE (n = 6 per each group). *P < 0.05; **P < 0.01 (Wilcoxon signed rank test).

To analyze the changes in the feeding pattern in more detail, we recorded the electromyography (EMG) activities of the masticatory muscles, i.e. the jaw opener digastric and jaw-closer masseter muscles, during nighttime feeding. The EMG activity of both the digastric and masseter muscles consists of two distinct patterns of bursts corresponding to the jaw movements in the gnawing phase, reflecting the cutting and intake performed with the front teeth, and in the chewing phase, reflecting the crushing by the molar teeth. As shown in Fig. 7A, the number of bursts in a fixed time period was smaller in the OSM rats than control rats, i.e., reduced from four to three in the gnawing phase and from six to five in the chewing phase, indicating slow eating in the OSM rats. As shown in Fig. 7B, in the OSM rats, the magnitude of the EMG bursts became smaller in the gnawing phase in the digastric muscle and the chewing phase in the masseter muscle, indicating weaker masticatory force in both the gnawing and chewing phases. A further analysis of discharge bursts in the masseter muscle confirmed these findings by the prolonged burst duration in both the gnawing and chewing periods (Fig. 7Ca) and the elongated inter-burst interval in the gnawing phase (Fig. 7Cb). Consequently, the mean chewing cycle in the OSM rats was significantly decreased in both phases (Fig. 7Cc). The milk rats showed very similar EMG patterns as in the control rats, except for the small burst duration (Fig. 7Ca) and the increased burst frequency (Fig. 7Cc) in the gnawing phase.

## Discussion

One essential finding in the present study is that the OSM odor down-regulates the prepro-orexin mRNA, indicating a lowered production of orexin in the hypothalamus after exposure to the OSM odor. It is reported that the prepro-orexin mRNA level is correlated with the product of orexin^22^. The present study revealed that the number of orexin-immunoreactive neurons in the lateral hypothalamus was significantly decreased by the OSM odor in comparison with the non-odor control and also the milk odor. The down-regulation of the prepro-orexin mRNA was maximum at 60 min after the onset of odor stimulation with OSM or milk. These down-regulation patterns are induced by the olfactory neural information transmitted from peripheral olfactory receptor cells to the brain, and not a humoral effect of the odor molecules absorbed from the respiratory mucosa to the brain, because the artificially induced dysfunction of the olfactory receptor cells in the nasal mucosa abolished the possibility of such odor effects.

It is well established that orexin is important in the maintenance of energy homeostasis, which is kept in balance by the storage of energy through food intake and by the expenditure of energy through body movement^23^. An imbalance of energy homeostasis can lead to either obesity or leanness. Since orexin increases food intake and arousal level^24^, orexin-KO mice eat less food and exhibit severely reduced spontaneous activity, eventually leading to obesity because the latter effect overcomes the former effect^25^,^26^,^27^. These findings strongly suggest that OSM odor plays a role in the regulation of food intake and arousal level.

Another important finding in the present study is that the odor of OSM decreased the expression of other orexigenic neuropeptides, such as MCH, AgRP and NPY, as well as orexin, and increased anorexigenic neuropeptides, such as CART and POMC. The odor of milk, on the other hand, decreased the levels of AgRP and NPY, but increased MCH and orexin, and had no effects on CART and POMC. It is interesting that the odor of milk has the opposite actions to orexin and MCH in the lateral hypothalamus^24^,^28^ compared with that of AgRP and NPY in the arcuate nucleus^29^,^30^, indicating that the differential effects of the odor of milk compared to that of OSM in the hypothalamus and the functional difference between orexin/MCH and AgRP/NPY among the orexigenic neuropeptides^31^.

The overall decrease of the expression of the orexigenic neuropeptides and increase of the anorexigenic neuropeptides by the OSM odor may explain the reduction of short-term and long-term food intake by this odor. The present study also showed that weight gain was moderately depressed by the odor of OSM in the long-term feeding test using young rats, indicating that the OSM odor induces a reduction of food intake more effectively than it induces a reduction of energy expenditure. Similar results were obtained in another experiment using young rats in which the odor and food were presented separately, but not mixed with food as in the present study, to exclude a possible post-ingestive effect of the ingested odorants on food intake^32^. That is, the odor of OSM was presented to the experimental rats by putting an OSM-soaked filter paper in the cage and they were allowed to eat pellets and drink water freely, and the only difference in the control rats was that the filter paper was soaked in distilled water. The experimental rats showed a gradual decrease in body weight along with the decreased food intake in comparison with the control rats and a significant difference was observed on the 27^th^ day after the start of experiment. In the present study, a significant difference was detected on the 10^th^ day. What is the reason for these differences? If the odorous food was unpleasant or stressful, the rats would be expected to curtail their intake on the first exposure, but they exhibited a similar intake until the 10^th^ day. Although an effect of the ingested OSM cannot be excluded, the retronasal olfactory stimulation during food intake in addition to the orthonasal stimulation appears to be more effective than the orthonasal stimulation by itself^33^,^34^.

As far as the relationship of odors to food and ingestion behavior is concerned, some previous researchers have accounted for odor effects by autonomic nervous system^35^,^36^,^37^,^38^,^39^. For example, Nagai and his colleagues^10^,^37^,^38^ showed that the flavor of grapefruit and its active component, limonene, increased and decreased activity of the sympathetic and parasympathetic nerves^37^, respectively, and reduced food intake and body weight^37^. In contrast, olfactory stimulation with the scent of lavender oil and its active component, linalool, had the opposite effect on the autonomic nervous system and food intake^11^,^39^. Concerning the OSM odor, previous studies^40^,^41^ reported that it activated the parasympathetic rather than sympathetic nervous system. It is of great interest that although both the OSM odor and grapefruit odor decrease food intake, their effect is opposite in terms of the autonomic nervous activation. The suppression of the orexigenic neuronal activity may be more dominant than the parasympathetic anabolic action in the case of the OSM odor.

To determine whether reductions of both food intake and body weight in rats exposed to the OSM odor are due to changes in the levels of the feeding-related neuropeptides, we did the same behavioral experiment in *ob/ob* mice with defective leptin signaling where orexin, MCH, NPY and AgRP are highly expressed^42^,^43^,^44^ and CART and POMC are depressed^42^. These mice did not show reductions of both food intake and body weight, suggesting that the OSM odor was not powerful enough to regulate the highly deviated levels of feeding-related neuropeptides in leptin-deficient animals. The tendency of gaining more body weight in the mice exposed to OSM than control *ob/ob* mice, although the difference was not statistically significant (see Fig. 5), might reflect the parasympathetic anabolic action of the OSM. Thus, we could demonstrate that lower mRNA levels for orexin, MCH, NPY and AgRP and high levels for CART and POMC are required for the OSM odor effects to occur.

Since the odor of OSM decreases the level of orexin along with other orexigenic peptides and orexin is important in elevating the arousal state, there is a possibility that the decreased food intake and reduced body weight were simply due to the lowered appetite resulting from a drowsy state without an effect on normal masticatory movements. To examine this possibility, we analyzed the feeding pattern and electromyography (EMG) data on the masticatory muscles during feeding with or without exposures to the OSM or milk odors. The results showed that the cumulative food intake was smaller in rats exposed to the odor of OSM than in rats exposed to the odor of milk. The latency to eat and the feeding time for a fixed amount of pellets were longer in the OSM than milk rats. EMG recordings from both the diagastric (jaw opening) and masseter (jaw closing) muscles showed two distinct patterns of bursts corresponding to the gnawing and chewing phases. In the OSM rats, the integrated EMG activity of both muscles became smaller in both the gnawing and chewing phases, the duration became longer in the chewing phase, the interval became longer in the gnawing phase and the frequency was decreased in the chewing phase compared with the milk rats. Thus, OSM-induced depressive feeding behavior is characterized by sluggish masticatory movements. Such low appetite and weak mastication in OSM rats are in contrast with the milk rats, and opposite to our recent finding^15^ that an intracerebroventricular injection of orexin-A facilitated feeding behavior, with powerful jaw-closer activities for the purpose of crushing the large amount of food taken into the mouth, like the case with human binge eaters.

Among the complex components of OSM, the essential ones include γ-decalactone, β-ionone, dihydro-β-ionone and the linalool oxides^45^,^46^. Which of these components are responsible for the feeding effects, either alone or in combination, is a subject for future study. The milk odor used in the present study also consists of various components, such as dipropylene glycol and delta-dodecalactone (based on a gas-chromatography analysis by Dr. R. Komaki, personal communication). There is thus a possibility that some of these components increase orexin and MCH expression in the lateral hypothalamic area, while the others decrease AgRP and NPY and increase CART and POMC expression in the arcuate nucleus of the hypothalamus.

In conclusion, the odor of OSM, which is known to have a mildly sedative effect, has two opposing effects on food intake: down-regulation of the mRNAs for the orexigenic neuropeptides together with up-regulation of the anorexigenic neuropeptides and facilitation of parasympathetic nerve activity. The present finding that the OSM odor attenuates food intake suggests that the former action overcomes the autonomic action. OSM may prove to be useful to control the energy balance of the body in terms of the prevention of over-eating and gaining weight. To the best of our knowledge, this is the first paper that reports the effects of odor stimulation on the expression of feeding-related neuropeptides.

## Methods

### Animals

In total, 163 Wistar male rats and five-week old male obese *ob/ob* (B6. Cg-*Lep^ob^*/J, n = 8) mice from Charles River, Inc. were used. Animals were individually housed in standard plastic cages in a temperature- and humidity-controlled room (23°C, 60%) on a 12:12 h light: dark cycle (lights on from 07:00 to 19:00). Food and water were available *ad libitum* except where noted. All animals were handled in accordance with the procedures outlined in the Guide for the Care and Use of Laboratory Animals (National Institute of Health Guide), and approval for this study was obtained from the institutional committee on animal research (Animal Research Committees of Osaka and Kio Universities).

### Olfactory stimuli

The essential oils of milk and Osmanthus fragrans (supplied by R. Komaki of the Seisyo Aroma Institute, Kanagawa, Japan) were used as olfactory stimuli. A drop (100 μl) of either one of the oils was put on a filter paper (3 × 3 cm), which was put between two metal mesh plates. The filter paper and plates were clipped and placed on the center of the chamber floor before the start of the experiment.

### Tissue sampling

The brains were quickly removed and placed in chilled brain matrix (Bioanalytical Systems, USA). A 3-mm thick coronal section was made with the caudal optic chiasma as the anterior boundary. The section was placed with the rostral surface facing upwards on a plate on ice. The hypothalamus was cut out, collected in a 1.5-ml sample tube, and preserved at −80 degrees.

### Quantitative RT-PCR

Since the semi-quantitative RT-PCR experiments raised the possibility that the smell of OSM influences the expression of prepro-orexin mRNA, we estimated the effects of OSM on prepro-orexin, MCH, AgRP and NPY as orexigenic peptides, and CART and POMC as anorexigenic peptides. To examine the changes in the expression of mRNA more precisely, we used a quantitative real time (RT)-PCR technique. RT-PCR assay was performed using a LightCycler® ST300 system (Roche Diagnostics K.K., Tokyo, Japan) according to the manufacturer's instructions. The reaction was carried out with QIAGEN QuantiTect SYBR Green PCR Master Mix in a 20 μL volume containing 0.5 μM of primers. A typical protocol, which took approximately 15 min to complete, included a 30-second denaturation step, followed by 35 cycles with a denaturation step for 5 s at 95°C, annealing for 5 s at 58°C, and extension for 5 s at 72°C. The extension periods varied with the specific primers, depending on the length of the product. An amplification curve was generated after analyzing the raw data and adjusting the threshold cycle (Ct) value, and a standard curve was acquired to calculate the unknown quantity. We tested each sample 3 times. The expression levels of mRNA are indicated as the relative cycle number normalized by the cycle number of GAPDH using LightCycler Relative Quantification Software (Roche Diagnostics). The primer set for GAPDH mRNA was 5′-GTCTTCACCACCATGGAGAAG-3′ (forward) and 5′-GCCAAAGTCATCCATGACAAC-3′ (reverse).

### Anosmic procedure

It is possible that stimulation with flavors induces not only olfactory perception but also absorption of the extract material by the lung into the bloodstream. To elucidate whether such material in the blood affects the expression of prepro-orexin mRNA, we performed an anosmic experiment^47^.

The rats that were maintained on the restricted food schedule were trained to perform in a buried peanut butter mash test designed to evaluate olfactory capacity. The peanut butter mash was a mixture of peanut butter (Skippy®Creamy, Unilever, NJ, USA) with powdered chow (MF, Oriental Yeast Co., Ltd., Tokyo, Japan). Each rat was placed in an open field (55 × 35 × 30 cm) and the floor of the apparatus was covered with approximately (7 cm) of sawdust (soft chip, Japan SLC, Inc., Shizuoka, Japan). Initially, the peanut butter mash sample was placed on top of the bedding until the rats consistently ate them. Subsequently, the peanut butter mash was buried beneath the bedding in random locations, and the latency to locate and consume the peanut butter mash was recorded. Each rat was removed from the apparatus either after the complete consumption of the peanut butter mash or at the end of the 5-min period.

Once all of the rats were consistently finding and consuming the peanut butter mash in less than 5 min, they were treated for the first time with zinc sulfate. The rats were lightly anesthetized with ether. They were placed on their backs with the head lowered. Zinc sulfate (50 mg/ml in 0.5% NaCl) (Anosmia group) or saline (Sham group) was infused into the nasal cavity. Excess fluid was removed from the oral cavity by first briefly holding the rat upside down and then by aspiration.

Disrupted olfactory capacity was confirmed using the peanut butter mash test on each of 2 days following the treatment with zinc sulfate. A rat was considered to be anosmic when it failed to locate and consume the peanut butter mash in less than 5 min. On the day after the above-mentioned odor presentation, tissue sampling was performed.

### Orexin expression in the neurons of the hypothalamus

To compare the changes in the expression of orexin in the hypothalamus between the OSM and milk odors, rats were perfused with saline followed by paraformaldehyde and the brain was removed 60 min after the onset of odor stimulation. Orexin was immunostained using a conventional immunohistochemical method^48^. A camera lucida was used to draw a horizontally- oriented (1700 μm × 1000 μm) rectangle that covered the dorsal and lateral hypothalamus, with the fornix located in the central region of the rectangle^49^,^50^. This rectangle was divided vertically into three regions in order to capture the medial, central and lateral areas (Fig. 3). The orexin-immunoreactive neurons were counted on one side of the brain for each of the three areas with the aid of a computer-assisted imaging analysis system (Image J, NIH, USA).

### Food intake and body weight over 18 days

To examine the effects of chronic exposure to the OSM odor on body weight, food intake and water consumption, rats were given mash scented with the OSM odor. The mash was a mixture of powdered chow with water in which an extract of OSM was diluted (0.25%). The weight of the powdered chow was equal to that of the water. Fresh mash was given to each rat every evening. At the same time, the body weight, intake of the mashes and consumption of water were measured.

### Food intake and body weight over 23days in *ob/ob* mice

To examine the effects of chronic exposure to the OSM odor on body weight, food intake and water consumption, *ob/ob* mice were given scented mash with or without the OSM odor for 23 days. The fresh mash was prepared and given to each mouse every evening as described above. At the same time, the body weight, intake of the mashes and consumption of water were measured.

### Masticatory muscle activity

To analyze the changes in the feeding pattern in more detail, we recorded the electromyography (EMG) activities of the masticatory muscles, i.e. the jaw opener digastric and jaw-closer masseter muscles during feeding in the nighttime. The methods employed in the EMG recording and analyses are described in our previous report^15^.

## Author Contributions

T.Y. and T.T. designed the entire study protocol. T.I. performed the molecular biological experiments with PCR and the behavioral experiment with chronic exposure to odors. T.T. performed the behavioral, immunohistochemical and electromyographic studies with acute exposure to odors. T.I. and T.T. performed the statistical analyses and interpretation of the results under the supervision of T.Y., T. Y. and T.T. wrote the manuscript, which all authors read and approved. These authors contributed equally to this work.
